# Recent Myocardial Infarction and Acute Heparin-Induced Thrombocytopenia: A Case Report on Perioperative Management of a Patient for Acute Limb Vascular Surgery

**DOI:** 10.7759/cureus.30763

**Published:** 2022-10-27

**Authors:** Sara Nogueira, Joana Queijo, Ana Rita Teles

**Affiliations:** 1 Department of Anaesthesiology, Centro Hospitalar Universitário de São João, Porto, PRT; 2 Department of Anaesthesiology, Centro Hospitalar de Leiria, Leiria, PRT

**Keywords:** perioperative period, acute limb ischaemia, acute myocardial infarction, argatroban, vascular surgery, heparin induced thrombocytopenia

## Abstract

The optimal alternative anticoagulation regimen for patients with heparin-induced thrombocytopenia (HIT) who need surgical procedures that involve higher levels of anticoagulation, usually performed under heparin, is not yet established. Argatroban has been reported as a safe alternative. Plasma levels and their anticoagulant effects follow a predictable profile. Also, it is easily monitored and its pharmacokinetic profile makes it suitable for patients with renal failure, as it undergoes hepatic elimination. However, its use as a substitute for heparin in HIT patients is not well-documented, especially in vascular surgery. We present a case of a successfully managed patient with acute HIT undergoing limb vascular surgery using anticoagulation with argatroban.

## Introduction

Unfractionated heparin (UFH) is frequently prescribed and routinely used for therapeutic and prophylactic anticoagulation in a variety of medical and surgical conditions [1]. Although hemorrhagic events are the most common complication, thrombotic events may also occur in patients who develop heparin-induced thrombocytopenia (HIT) [1-3]. HIT is a prothrombotic disorder that requires the discontinuation of heparin and the implementation of an alternative anticoagulation regimen [1-3]. Anticoagulation management of surgical patients with HIT is not yet well-established. Bivalirudin, argatroban, or danaparoid are alternative options. However, their use is not well-documented, especially in vascular surgery [2,4,5]. The choice of agent must account for drug and patient features and the experience of the clinician [2].

We present a case of a successfully managed patient with acute HIT undergoing limb vascular surgery using anticoagulation with argatroban.

## Case presentation

A 71-year-old male, American Society of Anaesthesiologists physical status ASA IV, with right lower acute limb ischemia and new-onset HIT was submitted to vascular surgery. He was first admitted to the emergency department with chest pain, being diagnosed with acute myocardial infarction of the inferior wall complicated by ventricular septal rupture. He was immediately submitted to primary percutaneous coronary intervention (PCI) with the placement of an intra-aortic balloon pump through the right femoral artery. After the procedure, intravenous perfusion of UFH was initiated. On the eighth day of his hospital stay, he was submitted to surgical correction of the ventricular septal defect. The immediate postoperative period was complicated by acute renal failure with the need for renal replacement therapy (RRT) and acute ischemia of the right lower limb. During the immediate evaluation by a specialist in vascular surgery, he had no sensory loss or motor deficit. Echo Doppler of the right lower limb showed extensive arterial calcification with an occlusive plate in the distal third of the superficial femoral artery, with a monophasic Doppler in the popliteal and posterior tibial arteries and inaudible Doppler in the dorsalis pedis artery. According to the Rutherford classification for acute limb ischemia, he had a grade IIA (marginally threatened) with no need for immediate revascularization. The initial appropriate medical treatment included analgesia and intravenous administration of UFH. On day 10, the patient developed a sudden drop in his platelet count, from 126 to 21 x 10^9^/L platelets. He had a high probability 4Ts score for HIT, with a platelet count fall superior to 50% and a platelet nadir superior to 20 x 10^9^/L, a clear thrombocytopenia onset between days 5 and 14, and no other causes of thrombocytopenia. The heparin-induced platelet activation assay was positive for platelet factor four antibodies, making the diagnosis of HIT. Due to the recent development of acute renal failure under RRT and the absence of hepatic impairment, UFH was stopped and a continuous intravenous infusion of argatroban was initiated at 2 mcg/kg/min. Therapy was monitored using activated partial thromboplastin time (aPTT) with a target range of 1.5 to 3 times the initial baseline value. Platelet count gradually increased to 204 x 10^9^/L. Table 1 presents the preoperative laboratory investigation results.

**Table 1:** Preoperative laboratory investigation results

Investigation	Patient value	Reference value
Haemoglobin (g/dL)	9.2	13.0-18.0
Leucocyte count (x10^9^/L)	13.29	4.0-11.0
Platelet count (x10^9^/L)	204	150-400
Alanine transaminase (U/L)	7	10-37
Aspartate transaminase (U/L)	57	10-37
Alkaline phosphatase (U/L)	88	30-120
Gamma-glutamyl transferase (U/L)	45	10-49
Creatinine (mg/dL)	2.89	0.67-1.17
Sodium (mEq/L)	136	135-147
Potassium (mEq/L)	4.5	3.5-5.1
C-reactive protein (mg/L)	66.0	<3.0

**Figure 1:**
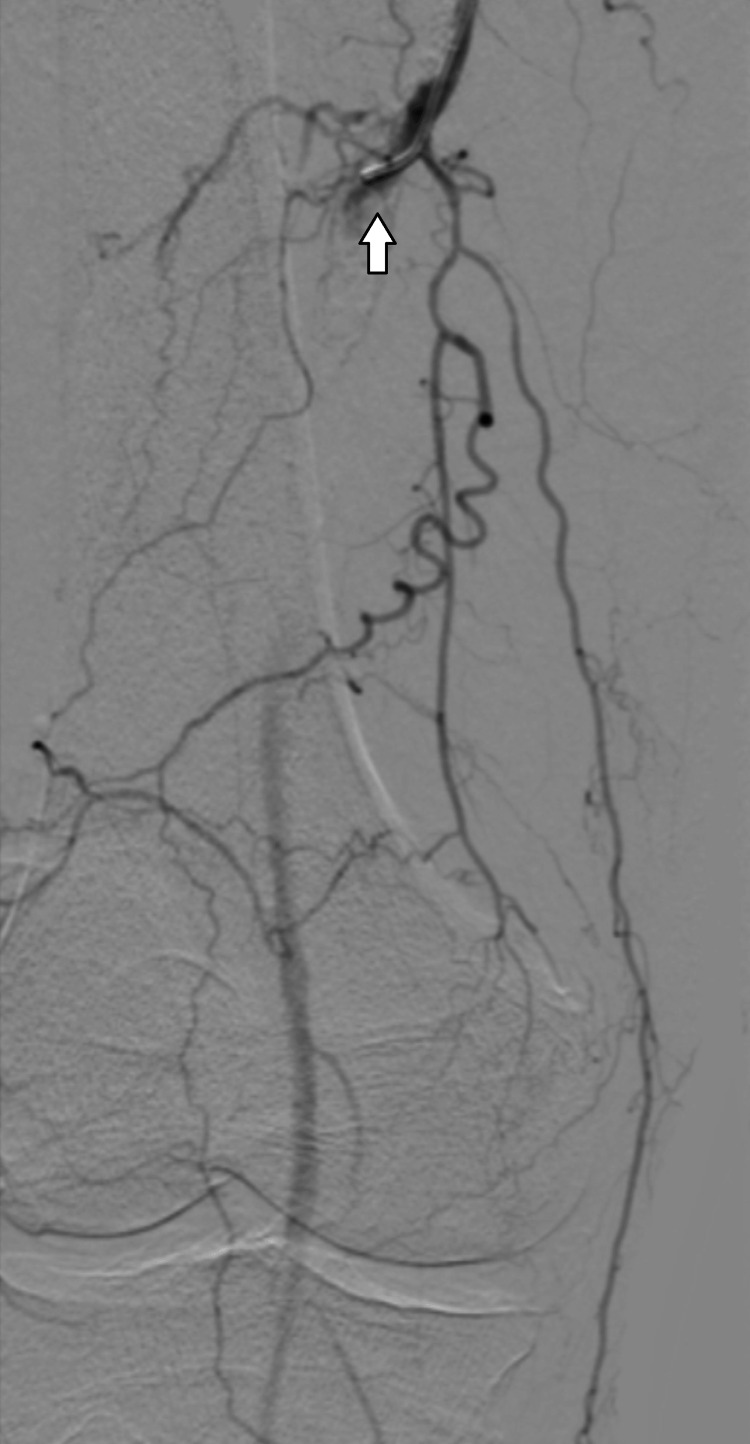
Digital subtraction angiography showing occlusion of the right superficial femoral artery

On the 19th day of the hospital stay, he was submitted to percutaneous transluminal angioplasty of the right superficial femoral artery. Figure 1 shows the occlusion site.

**Figure 2:**
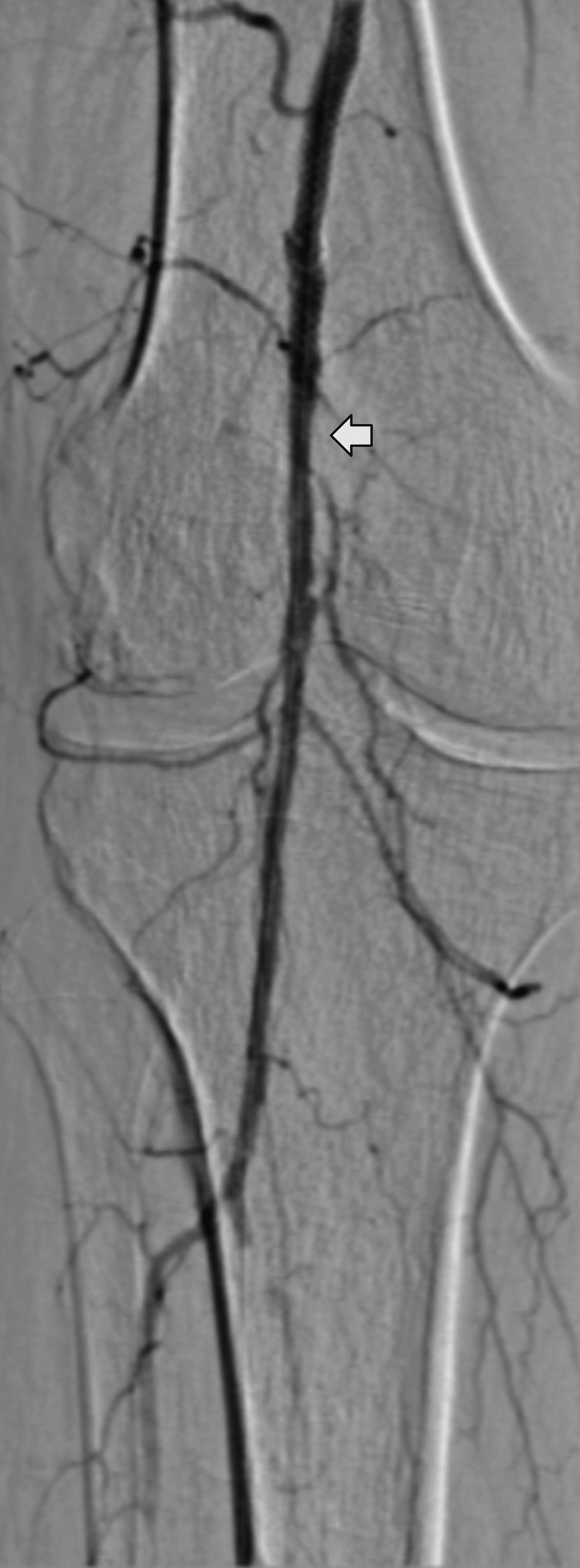
Digital subtraction angiography showing limb revascularization

The procedure was performed under sedation with dexmedetomidine intravenous infusion (0.7-1.4 mcg/kg/h). Before the beginning of the endovascular procedure, continuous intravenous infusion of argatroban was adjusted to 25 mcg/kg/min, and after 5-10 minutes, the initial activated clotting time (ACT) was 215 seconds. An ACT of ≥ 300 seconds was achieved using an intravenous argatroban bolus dose of 150 mcg/kg and an increase of the infusion dose to 30 mcg/kg/min. An additional bolus of 150 mcg/kg was administered to maintain an ACT of ≥ 300 seconds. The intraoperative period was uneventful, with no hemodynamic instability and minimal blood loss. Lower limb revascularization, as shown in Figure 2, was achieved at the end of the procedure.

Immediate postoperative surveillance was achieved in the postanesthesia care unit. As anticoagulation was required after the procedure, a new aPTT was obtained two hours later, and the argatroban infusion rate was reduced to maintain an aPTT with a target range of 1.5 to 3 times the initial baseline value. Anticoagulation monitoring with aPTT was performed two hours after every change in infusion rate. The therapeutic range was achieved at 3.6 mcg/kg/min. Surgery was successfully performed without any thromboembolic or bleeding complications. Table 2 summarizes the doses and monitoring performed during the entire perioperative period.

**Table 2:** Doses and monitoring of argatroban during the perioperative period aPTT, activated partial thromboplastin time; ACT, activated clotting time; s, seconds ^1^ aPTT measured two hours after dose adjustment ^2^ ACT rechecked 5-10 minutes after dose adjustment

Perioperative period	Argatroban dose	Monitoring
Preoperative period	Infusion rate: 2 mcg/kg/min	aPTT^1^: 1.5 to 3 times the initial baseline value
Intraoperative period	(↑)Infusion rate: 25 mcg/kg/min	ACT^2^: < 300 s
	(=)Infusion rate: 25 mcg/kg/min + Bolus: 150 mcg/kg	ACT^2^: < 300 s
	(↑)Infusion rate: 30 mcg/kg/min + Bolus: 150 mcg/kg	ACT^2^: < 300 s
	(=)Infusion rate: 30 mcg/kg/min + Bolus: 150 mcg/kg	ACT^2^: ≥300 s
Post-operative period	Infusion rate: 4.5 mcg/kg/min	aPTT^1^: > 1.5 to 3 times the initial baseline value
	Infusion rate: 3.6 mcg/kg/min	aPTT^1^: 1.5 to 3 times the initial baseline value

## Discussion

HIT is an antibody-mediated adverse drug reaction to UFH, and less commonly to low-molecular-weight heparin. It is characterized by a 50% fall in platelet count from baseline, and it usually occurs 5-14 days after initial exposure. Its prevalence ranges from 0.1% to 5.0%, and has a reported mortality between 20-30% [1-3]. Despite rare, it is a lethal syndrome caused by antibodies against complexes of platelet factor four and heparin (HIT antibodies) [5]. The diagnosis involves both clinical and laboratory components [1]. The 4Ts score system is the most commonly used and if there is an intermediate- or high-probability 4Ts score, an immunoassay is recommended [2]. Although characterized by thrombocytopenia, the disease results in a paradoxical prothrombotic disorder and arterial and venous thromboembolic events may occur [1,3,4].

It is recommended that surgical procedures routinely performed under heparin should be delayed in patients with HIT until the patient has subacute or remote HIT. However, if surgery cannot be delayed, the optimal alternative anticoagulation regimen should be individualized [2,6].

Argatroban is a synthetic, reversible, direct thrombin inhibitor that forms a univalent binding to the active site. It inhibits both plasma and fibrin-bound thrombin, with the former being responsible for platelet activation and clot stabilization. Thus, argatroban decreases clot stabilization, promotes thrombolysis, and increases the fibrin network permeability, rendering it less resistant to fibrinolysis [7,8]. Upon initiation of continuous infusion, plasma levels and anticoagulant effects follow a predictable profile. Steady-state plasma concentrations increase proportionally and are well correlated with steady-state anticoagulant effects. Its pharmacokinetic profile makes it suitable for patients with renal failure, as it undergoes hepatic elimination with a half-life of 40 to 50 minutes [7,8]. In the presented case, as the patient developed acute renal failure with the need for RRT, argatroban was the most suitable choice.

For acute HIT, the recommended initial dose of argatroban for adult patients without hepatic impairment is 2 mcg/kg/min. It is administered as a continuous infusion and monitored using aPTT with a target range of 1.5 to 3 times the initial baseline value. Dose adjustment, until a maximum of 10 mcg/Kg/min, should be made to obtain a steady state. aPTT two hours after initiation and any dose adjustment should be obtained to confirm the desired therapeutic range [6-8].

In the setting of procedures that involve higher levels of anticoagulation argatroban has been reported as an alternative to heparin in HIT patients. In the particular case of cardiac surgery, left ventricular assist device implantation, PCI, and arterio-venous extracorporeal membrane oxygenation support there is a limited number of smaller prospective and retrospective studies [7-10]. In the perioperative period, argatroban anticoagulation monitoring is performed with ACT, a point-of-care test with a fast turnaround time, for a target range of 300-450 seconds. Accordingly, with most of the available literature, as was done in the presented case, the target value was achieved by continuous infusion along with bolus dose and ACT rechecked 5-10 minutes later [7-10].

In the particular case of vascular surgery literature and experience are lacking. When patients with a prior history of HIT or acute HIT undergo peripheral vascular surgery, argatroban, at a dose determined by ACT, seems a viable and safe substitute for UFH. Because it does not resemble heparin, there is no cross-reactivity with HIT antibodies. It is easy to monitor, with little potential for over or underdosing, regardless of renal function, making it reliable in patients with renal dysfunction. However, the lack of a reversal agent must be considered a potential hazard and challenge. This limitation must be considered when trying to balance the potential risks and benefits of argatroban [4,8].

## Conclusions

Heparin-induced thrombocytopenia is a rare but potentially life-threatening syndrome that requires an alternative anticoagulation regimen. The perioperative management of these patients scheduled for surgeries that are routinely performed under unfractionated heparin is challenging, as there are no established alternative regimens. Literature is scarce, especially for vascular surgery, so this case report brings more detailed insight into this field with applicable results.

## References

[1] Salter BS, Weiner MM, Trinh MA, Heller J, Evans AS, Adams DH, Fischer GW (2016). Heparin-induced thrombocytopenia: a comprehensive clinical review. J Am Coll Cardiol.

[2] Hassell K (2008). Heparin-induced thrombocytopenia: diagnosis and management. Thromb Res.

[3] Tokuda Y, Matsumoto M, Sugita T, Nishizawa J, Matsuyama K, Yoshida K, Matsuo T (2003). Vascular surgery using argatroban in a patient with a history of heparin-induced thrombocytopenia. Circ J.

[4] Björck M, Earnshaw JJ, Acosta S (2020). Editor’s choice - European Society for Vascular Surgery (ESVs) 2020 clinical practice guidelines on the management of acute limb ischaemia. Eur J Vasc Endovasc Surg.

[5] Cuker A, Arepally GM, Chong BH (2018). American Society of Hematology 2018 guidelines for management of venous thromboembolism: heparin-induced thrombocytopenia. Blood Adv.

[6] Lewis BE, Wallis DE, Berkowitz SD (2001). Argatroban anticoagulant therapy in patients with heparin-induced thrombocytopenia. Circulation.

[7] (2022). Argatroban. https://www.accessdata.fda.gov/drugsatfda_docs/label/2011/022485lbl.pdf.

[8] Koster A, Faraoni D, Levy JH (2018). Argatroban and bivalirudin for perioperative anticoagulation in cardiac surgery. Anesthesiology.

[9] Rössig L, Genth-Zotz S, Rau M (2011). Argatroban for elective percutaneous coronary intervention: the ARG-E04 multi-center study. Int J Cardiol.

[10] Van Cott EM, Roberts AJ, Dager WE (2017). Laboratory monitoring of parenteral direct thrombin inhibitors. Semin Thromb Hemost.

